# High-Resolution Magic-Angle Spinning NMR Spectroscopy for Evaluation of Cell Shielding by Virucidal Composites Based on Biogenic Silver Nanoparticles, Flexible Cellulose Nanofibers and Graphene Oxide

**DOI:** 10.3389/fbioe.2022.858156

**Published:** 2022-05-12

**Authors:** Danijela Stanisic, Guilherme C. F. Cruz, Leonardo Abdala Elias, Junko Tsukamoto, Clarice W. Arns, Douglas Soares da Silva, Stanislav Mochkalev, Raluca Savu, Ljubica Tasic

**Affiliations:** ^1^ Chemical Biology Laboratory, Department of Organic Chemistry, Institute of Chemistry, University of Campinas (UNICAMP), São Paulo, Brazil; ^2^ Centre for Semiconductor Components and Nanotechnology (CCSNano), University of Campinas (UNICAMP), São Paulo, Brazil; ^3^ Center for Biomedical Engineering (CEB), University of Campinas (UNICAMP), São Paulo, Brazil; ^4^ Department of Electronics and Biomedical Engineering, School of Electrical and Computer Engineering, University of Campinas (UNICAMP), São Paulo, Brazil; ^5^ Laboratory of Animal Virology, Department of Genetics, Evolution, Microbiology, and Immunology, Institute of Biology, University of Campinas (UNICAMP), São Paulo, Brazil; ^6^ Institute of Chemistry, University of Campinas (UNICAMP), São Paulo, Brazil

**Keywords:** silver nanoparticles, hesperetin, graphene oxide, cellulose nanofibers (CNF), composites, virus, HR-MAS ^1^H-NMR spectroscopy

## Abstract

Antiviral and non-toxic effects of silver nanoparticles onto *in vitro* cells infected with coronavirus were evaluated in this study using High-Resolution Magic-Angle Spinning Nuclear Magnetic Resonance (HR-MAS NMR) spectroscopy. Silver nanoparticles were designed and synthesized using an orange flavonoid—hesperetin (HST)—for reduction of silver(I) and stabilization of as obtained nanoparticles. The bio-inspired process is a simple, clean, and sustainable way to synthesize biogenic silver nanoparticles (AgNP@HST) with diameters of ∼20 nm and low zeta potential (−40 mV), with great colloidal stability monitored for 2 years. The nanoparticles were used for the fabrication of two types of antiviral materials: colloids (AgNP@HST spray) and 3D flexible nanostructured composites. The composites, decorated with AgNP@HST (0.05 mmol L^−1^), were made using cellulose nanofibers (CNF) obtained from orange peel and graphene oxide (GO), being denominated CNF@GO@AgNP@HST. Both materials showed high virucidal activity against coronaviruses in cell infection *in vitro* models and successfully inhibited the viral activity in cells. HR-MAS ^1^H-NMR technique was used for determining nanomaterials’ effects on living cells and their influences on metabolic pathways, as well as to study viral effects on cells. It was proven that none of the manufactured materials showed toxicity towards the intact cells used. Furthermore, viral infection was reverted when cells, infected with the coronavirus, were treated using the as-fabricated nanomaterials. These significant results open possibilities for antiviral application of 3D flexible nanostructured composite such as packaging papers and filters for facial masks, while the colloidal AgNP@HST spray can be used for disinfecting surfaces, as well as a nasal, mouth, and eye spray.

## Introduction

Due to their antimicrobial, i.e., anti-bacterial, -fungal, and -viral properties, silver(I) compounds, and silver(0) nanoparticles (AgNPs) have been extensively studied, mainly in the fields of biomedicine (Du et al., 2018; de Barros et al., 2018; Noor et al., 2019; de Faria et al., 2014; de Moraes et al., 2015; Wang et al., 2015; Sahu et al., 2016; Rathore et al., 2020). When used in low amounts, silver is non-toxic to humans (Abou Aitah et al., 2020). There are many possible applications for AgNPs as bioactive agents for a wide variety of viruses such as Human immunodeficiency virus type 1 (HIV-1), Influenza, Hepatitis B virus (HBV), Tacaribe virus (TCRV), Herpes simplex virus type 1 (HSV-1), Monkeypox virus, and Respiratory syncytial virus (Jones, 2020; Du et al., 2018; de Moraes et al., 2015; Abou Aitah et al., 2020). Mechanisms used by silver nanoparticles against virus replication and spread extent from inactivation of the virus before its interaction with the host cells, thus stopping its entry into the cell, to competing with virus binding to the cell. Some AgNPs may interfere in the viral attachment by blocking the virus binding and penetrating the host cell (Matharu et al., 2020; Innocenzi and Stagi, 2020; Du et al., 2018). Other mechanisms include interaction with the proteins, glycosides, or lipids on the virus surfaces and, subsequently, with the DNA or RNA in their interior (de Barros et al., 2018; Noor et al., 2019; Ye et al., 2015; de Faria et al., 2014; de Moraes et al., 2015; Wang et al., 2015; Sahu et al., 2016; Rathore et al., 2020; Abou Aitah et al., 2020). The AgNP bioactivity depends on their sizes, shapes, zeta potentials, and stabilizing agents covering the surface of the particles. For example, nanoparticles with diameters from 1 to 10 nm can attach to the HIV-1 in a rather regular spatial arrangement with the viral envelope and bind to the cysteine (-SH) residues of HIV-1 glycoprotein (Abou Aitah et al., 2020). Regarding the stabilizing agents, it is possible to functionalize nanoparticles with tailored capping that mimics the host cell virus receptors, thus substituting host-cell binding sites with the designed nanomaterial. If the core material, in this case—silver (0) nanoparticles, is nontoxic to the host cells, can be considered an ideal option as an antiviral (de Barros et al., 2018; Noor et al., 2019; de Faria et al., 2014; de Moraes et al., 2015; Wang et al., 2015; Abou Aitah et al., 2020; Socol et al., 2002; Zhu et al., 2000). This strategy has already been explored for viruses that use heparin sulfate-mediated cell entry. Most, but not all, antiviral AgNPs are in the 10–80 nm size range, where smaller ones exhibit more pronounced toxic effects on cells and human cell models. Since larger AgNPs can also exhibit cells toxicity, the best choice for an ideal antiviral activity would be nanoparticles within the 15–25 nm diameter range, with non-toxic and biocompatible agents capping their surfaces (Du et al., 2018; de Faria et al., 2014; de Moraes et al., 2015; Sahu et al., 2016; Rathore et al., 2020; Abou Aitah et al., 2020; Yazdanshenas and Shateri-Khalilabad, 2013).

Coronaviruses are spherical, highly organized, and tailored nanoparticles (around 100 nm) composted from lipids, proteins, and RNA, which infect animals and humans. The coronaviruses that caused Severe Acute Respiratory Syndrome (SARS), Middle East Respiratory Syndrome (MERS), and, more recently, COVID-19, are considered extremely harmful (Jones, 2020; Rose and Weiss, 2009; Matharu et al., 2020; Innocenzi and Stagi, 2020; Du et al., 2018). To obtain positive and disease-solving immune responses, the virus load must be maintained as low as possible (Jones, 2020). SARS-CoV-2, which provokes COVID-19, is unusually stable toward pH change and unstable when exposed to detergents. It is also omnipresent in the air and there are no efficient antiviral drugs for COVID-19 treatment (Jones, 2020; Rose and Weiss, 2009). Therefore, it is very important to design, develop and apply innovative virucidal nanomaterials that act in the early stages of infection, potentially stopping viral replication and the proliferation of the virus (Matharu et al., 2020; Innocenzi and Stagi, 2020; Du et al., 2018; de Barros et al., 2018; Noor et al., 2019; Ye et al., 2015; de Faria et al., 2014; de Moraes et al., 2015).

Inspired by the interesting and remarkable antiviral properties of the AgNP colloids, we designed a novel route for producing bio-based AgNPs exploring, for the first time, flavone isolated from orange peels as a reductive and stabilizing agent. The proposed bio-based process is clean, fast (almost instant), and yields very stable colloids.

The murine coronavirus (Mouse Hepatitis Virus—MHV-3) infection of the fibroblast L929 cell line was used as a model for this study (Cadagan and Merry, 2013). MHV-3 is a virus from the Coronaviridae family that counts on 31 Kb single-strand positive RNA genome replication in the cytoplasm of the infected cells. It is very similar to the SARS-CoV-2 and is recommended for studying infections with coronaviruses (Garcia et al., 2021; Rose and Weiss, 2009). Using this model, the AgNP@HST showed excellent bioactivity properties and virucidal effect, inhibiting coronavirus cell infection to a 99.9% extent.

Aiming to design and develop not only colloids as an effective virucidal nanomaterial, but 3D flexible composite nanomaterials were also fabricated and decorated with the AgNP@HST. For this, cellulose nanofibers (CNFs) were employed as one composite component, due to their excellent mechanical, absorptive, and physical properties, with graphene oxide being the other component (de Barros et al., 2018; de Faria et al., 2014; Rathore et al., 2020). Graphene oxide (GO) is known for its antiviral properties explored previously on DNA and RNA viruses (Matharu et al., 2020; Du et al., 2018; Ye et al., 2015; de Faria et al., 2014; de Moraes et al., 2015; Wang et al., 2015), and herein against coronavirus. The cellulose nanofibers with diameters of 22 nm, isolated from orange peels in an acid-base water extraction process, were used. Graphene oxide (GO) is a hydrophilic and virucidal nanomaterial, where the virus is expected to be stopped before reaching the host cell due to collision, adsorption, and electrostatic interactions with the negatively charged GO (Matharu et al., 2020; Du et al., 2018; Ye et al., 2015; de Faria et al., 2014; de Moraes et al., 2015; Wang et al., 2015). Thus, flexible 3D nanocomposites were fabricated in an easy four-step process, consisting of 1) preparation of nanomaterials’ suspension (CNF and GO), 2) mixing, homogenization, and filtering, 3) drying, and 4) decoration with the AgNP@HST. The fabricated material (CNF@GO@AgNP@HST) showed excellent mechanical properties, non-toxicity toward cells, and potent virucidal effects by almost complete neutralization of the virus titer (99.99%).

High-resolution magic angle spinning nuclear magnetic resonance (HR-MAS NMR) spectroscopy is applied as an analytical technique for the study of intact semi-solid biological samples such as cells and tissue (Stanisic et al., 2022; Vermathenet al., 2015; Henoumon et al., 2015). This technique acquires high-resolution NMR spectra, useful to identify small molecules and their pathways through metabolomics through metabolomics (Kim et al., 2011). Samples can be analyzed without prior processing or destruction, being the main advantage of this method over others. Intracellular metabolic variations and compositional changes of the cell culture on and in the cell are studied in different applications of nanoparticles. HeLa cells and their metabolic variation in interaction with silica nanoparticles is an example of NMR-based metabolic analyses (Feng et al., 2013). In this study, the high-resolution magic angle spinning ^1^H nuclear magnetic resonance spectroscopy (HR-MAS ^1^H NMR) was used for the evaluation of cells metabolites and cells infected with the virus, both in contact with the studied materials, namely AgNP@HST, GO, CNF, GO@AgNP-HST, and CNF@GO@AgNP@HST.

## Material and Methods

Hesperetin (2S)-5,7-dihydroxy-2-(3-hydroxy-4-methoxyphenyl)-2,3-dihydrochromen-4-one (>95%) (Supplementary Figure S1A), silver(I) nitrate, sodium hydroxide, and hydrogen chloride were purchased from Sigma Aldrich (St. Louis, United States). The cellulose nanofibers were obtained from the orange peels by sequential extraction in a three-step process: 1) removal of pectin and hemicelluloses, 2) bleaching the cellulose with hydrogen peroxide, and 3) nanonization of cellulose using an ultrasound [Mariño et al., 2021). Graphene oxide was prepared by the Hummers’ method (Yu et al., 2016).

### Silver Nanoparticles Synthesis and Characterization

Biogenic silver nanoparticles were synthesized using a 4-step process: 1) hesperetin was dissolved in alkaline solution (sodium hydroxide, NaOH, 0.005 mol L^−1^) to a final 1 mmol L^−1^ concentration, 2) a solution of silver (I) nitrate (1 mmol L^−1^) was added dropwise to a hesperetin solution in a 1:1 (v v^−1^) ratio, and then the 3) colloid pH of 7.4 was reached using a diluted solution of hydrochloric acid (HCl 0.05 mol L^−1^). The average hydrodynamic diameter of nanoparticles was determined by Dynamic Light Scattering (DLS) and the surface charge was measured in a Zetasizer Nano series equipment (Malvern Instruments). The potential was measured by electrophoretic mobility using dispersions of nanoparticles in a KCl (USB) solution at 1.0 mmol L^−1^ concentration and a Zetasizer Nano ZS analyzer (Malvern Instruments Corp., Malvern, United Kingdom). The AgNP@HST morphology was evaluated by Transmission Electron Microscopy (TEM). After 1:100 (v v^−1^) dilution of 0.5 mmol ml^−1^ of colloid silver nanoparticles in water, samples were deposited on carbon-coated film supported in 400 mesh copper grids (Ted Pella) and observed using a Libra 120 (Zeiss) microscope equipped with a spectrometer in-column “omega” and imaging system Olympus (OSIS), with Cantega G2 camera and iTEM software.

### 3D Flexible Nanostructured Composites Manufacture

The flexible 3D nanostructured composites were manufactured from cellulose nanofibers (CNFs), graphene oxide nanostructured powder (GO), and decorated with silver nanoparticles (AgNP@HST). The composites were obtained, by vacuum filtration, in the form of freestanding layers/sheets. The manufacture comprised: 1) the preparation of cellulose nanofibers. Cellulose nanofibers were added to the aqueous solution of sodium hydroxide (NaOH, Sigma-Aldrich), 7% by mass, and sonicated, in an ice bath (4°C), using a 7 mm probe (diameter of the tip) at 70% amplitude and 90 W (Hielscher, Model UP400ST) with a pulse of 2 s (Mariño et al., 2021). This mixture remained in the freezer for 1 h at 1.5°C; 2) Graphene oxide (GO) was suspended in deionized water by ultrasound for 10 min at room temperature (25°C); 3) AgNP@HST were added dropwise into the as-obtained GO suspension and sonicated for another 5 min or sprayed onto newly obtained GO@CNF after their mixture and filtration; 4) The suspensions of CNF and pristine GO or GO@AgNP@HST were mixed and filtered, leading to the manufacture of freestanding composites; and 5) GO@CNF papers decorated with AgNP@HST were obtained by vacuum filtration and 24 h drying at room temperature (25°C). The nanocomposite materials were characterized using Scanning Electron Microscopy (SEM, Dual Beam Nova 200 Nanolab, FEI) with Energy Dispersive X-ray Spectroscopy (EDS) for elemental analysis (Oxford Instruments). The mechanical tests were performed in a Universal Testing Machine (MTS, Alliance RT/5, 1000 N) following the ASTM D 882-02 and ASTM D 790 standards for tensile and flexure characterization, respectively. For tensile stress, a 10 mm min^−1^ speed was used, while a 5 mm min^−1^ test speed was applied for flexure.

### Cytotoxicity and Antiviral Activity

For cytotoxicity and viral inhibition analyses, fibroblast L929 cell lines (ATCC® CCL-1TM) were incubated in Dulbecco minimal essential culture medium (DMEM) supplemented with 10% fetal bovine serum (FBS) with 5% CO_2_ at 37° C. Subsequently, the L929 cells were seeded into 96-well plates (2 × 10^5^ cells wells^−1^ of density) were incubated, at 37° C with 5% CO_2_, with the obtained nanomaterials for observing their cytotoxicity effects. The cell viability was directly examined in an inverted microscope (quadruplicate). The antiviral activity (EN 14476: 201923, ASTM E1053-11) was assessed using the 50% endpoint method by serial dilution. This procedure consisted in making contact, at room temperature, between the strain murine hepatitis virus (MHV-3 Coronaviridae lineage (10^8^ mL^−1^, 500 µL) (Garcia et al., 2021), a surrogate for SARS-CoV-2, and the nanomaterials. Subsequently, the treated virus solution was serially diluted and transferred to confluent monolayer L929 cells. Once the incubation process (48 h in a 5% CO_2_ at 37°C) ended, the cytopathic effect of viral infection was verified and correlated with the cell and virus controls. Every assay was performed in quadruplicate. The virus titer was calculated using the Reed and Muench method (1938) (Reed and Muench, 1938). The results were expressed as a percentage of viral inactivation compared to untreated viral control (virus titer) in means ± standard deviation (SD). Each experiment tested a single sample formulation and replicated it four times. All data are reported as mean ± standard deviation (SD, Origin 8.1 SR3, v8.1.34.90, United States).

### Cell Preparation for NMR Experiments

Fibroblast L929 cell lines (ATCC® CCL-1TM) were grown as described in the previous section (2.3) in flasks to obtain at least 8 × 10^6^ cells mL^−1^ for each of the investigated conditions: 1) cells, 2) cells treated with the AgNP@HST (1:10 and 1:100, v v^−1^), 3) cells infected with the virus, and 4) infected cells treated with: 1) AgNP@HST (1:10 and 1:100, v v^−1^), 2) GO (1:10 and 1:100, v v^−1^), 3) GO@AgNP@HST (1:10 and 1:100, v v^−1^), and 4) CNF@GO@AgNP@HST (1:10 and 1:100, v v^−1^). The L929 cells adhered to the surface area of the bottom of the flasks were carefully prepared for loading into 4 mm, 12 µL rotors. The culture medium was removed, and the cells were washed with 8 ml of phosphate buffer saline (PBS, 2 mol L^−1^ NaCl, 2 mol L^−1^ KCl, 0.2 mol L^−1^ Na_2_HPO_4_, 1 mol L^−1^ KH_2_PO_4_). Next, a solution of trypsin in PBS (1:3, v v^−1^) was added and incubated for 1–2 min. After trypsinization, a culture medium was put into the flask to inactivate trypsin and prevent cell damage. The cell suspension was centrifuged at 1,500 rpm for 5 min (Eppendorf 5702) and the culture medium was carefully aspirated so that the pellet did not fracture. The pellet was then suspended in deuterated water and centrifuged (1,000 rpm for 5 min) and the samples for NMR analyses (metabolomic analysis) were obtained (Kaebiscg et al., 2017). A total of eight samples were prepared and characterized, using the same amount of cells for all pellets.

### HR-MAS ^1^H-NMR Analyses

HR-MAS ^1^H-NMR spectroscopy measurements were obtained at 9.4T (400.21 MHz for ^1^H), on a DRX 400 Bruker NMR spectrometer, using a spinning rate of 3 kHz, and a 4-mm triple resonance gradient HR-MAS probe. Sodium trimethylsilyl-[2,2,3,3-2H4]-1-propionate (TMSP) was used as an internal standard. The spectra were acquired with a presaturation pulse of 1.5 s, the acquisition time of 4.63 s (32k points), recycle delay of 4 s, and accumulation of 256 transients. In addition, before data acquisition, a Carr-Purcell-Meiboom-Gill (CPMG) spin-echo train was used by applying 120 cycles separated by 1.2 ms of echo time. The free induction decay (FID) signal was multiplied by a 1.0-Hz (0.0025 ppm) line-broadening factor, as well as a zero-filled two-fold for Fourier transform. 2D NMR ^1^H-^1^H TOCSY spectra were recorded with a Bruker/Topspin 3.0 DIPSI2 pulse sequence, the acquisition time of 232 ms, mixing time of 70 ms, spectral width of 11 ppm, and a relaxation delay of 3.0 s, with 64 scans. All spectra were processed and analyzed using the Topspin 3.0 software (Bruker BioSpin). Online databases (Human Metabolome Database, HMDB) were used for assignments.

## Results

**FIGURE 1:**
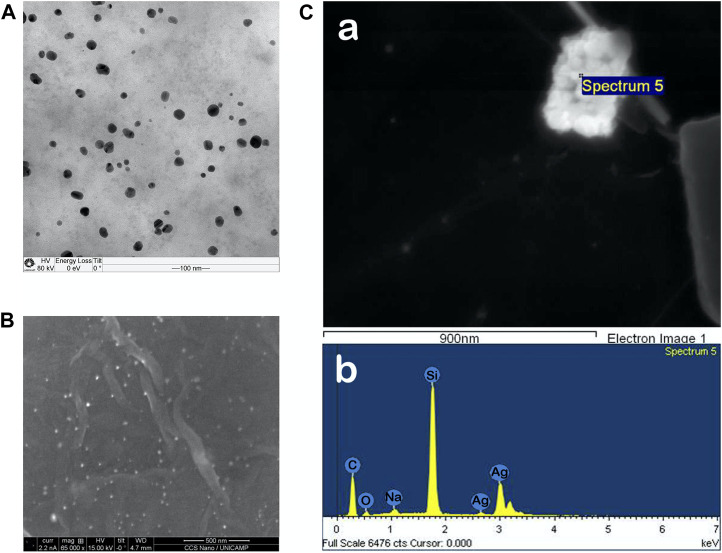
**(A)** TEM image of AgNP@HST showing the distribution of particles with 22 nm in diameter (scale bar = 100 nm). **(B)** SEM image of a CNF@GO@AgNP@HST composite decorated with AgNP@HST as 20 nm bright spots (scale bar = 500 nm) **(C)** GO sheet decorated with AgNP@HST particles (Spectrum 5) and its EDS graph.

Eco-friendly and bio-based silver nanoparticles were synthesized and tested against cell models for viral infection. Hesperetin, a natural polyphenol, was used for the synthesis and stabilization of the AgNP@HST. The formation of biogenic silver nanoparticles was quick, observed by an instant color change, from transparent to light orange, and monitored using UV-Vis measurements. Thus, the Plasmon band at 404.5 nm was observed. Ag(I) was reduced to the Ag(0) through oxidation of substituted aromatic rings of hesperetin, while non-consumed HST interacted with the formed AgNP and stabilized them to AgNP@HST (Figure 1). The nanoparticles showed mean diameters of 22 nm (Figure 1 and Supplementary Figure S1), a low Zeta potential of −40 mV, and a polydispersity index (PDI) below 0.3. The synthesized AgNP@HST colloid showed stability for at least 24 months.

The flexible nanostructured composites were formed from cellulose nanofibers (CNF, Supplementary Figure S2) and graphene oxide nanostructured powder (GO), decorated using *in* or *ex situ* processing, with silver nanoparticles (Supplementary Figure S3). The freestanding composite layers showed very good mechanical (Table 1) and virucidal (Table 2) properties. The use of CNF provided a better malleability and higher bending resistance for the GO freestanding films, with a uniform decoration by AgNP@HST (Figures 1B,C).

**TABLE 1:** Mechanical performance of the tested nanomaterials/composites.

Samples	Tensile modulus (GPa)	Flexural modulus (GPa)
CNF@AgNP@HST	0.41	12.6
GO@AgNP@HST	0.11	11.78
CNF@GO@AgNP@HST	0.52	14.0

**TABLE 2:** Toxicity data of the tested nanomaterials/composites.

	Cell line L929		
**Samples**	**Dilution v v^−1^**	**% Cytotoxicity**	**Cytotoxicity**
AgNP@HST	1:1	1.50	Not toxic
AgNP@HST	1:10	0.25	Not toxic
GO	1:10	2.50	Very low toxicity
GO@AgNP@HST	1:10	2.50	Very low toxicity
CNF@GO@AgNP@HST	1:10	0.25	Not toxic

AgNP@HST, bio-based silver nanoparticles; GO, graphene oxide; GO@AgNP@HST, nanocomposites obtained using graphene oxide decorated with the AgNP@HST; CNF@GO@AgNP@HST, 3D nanocomposites using CNF, GO, and AgNP@HST.

Regarding the mechanical resistance to tensile, it was observed an improvement of the Young modulus for composites based on CNF (Table 1). This can be due to the formation of a more resistant network provided by the cellulose nanofibers (Sun et al., 2010). Moreover, the mixtures of the two main mechanical components brought a higher resistance to flexure, preventing breaks with low tensions (10 mm maximum flexure used). Bending is exactly the mechanical deformation encountered when applying the composites as mask filters, indicating that, for this application, the mechanical properties of our composites are granted.

The fibroblast L929 cells infected with Murine Hepatitis Virus type 3 (Supplementary Figure S4) were used as a coronaviruses model system for studying the effects of the viral infection (Supplementary Figure S5). The designed nanomaterials were tested for toxicity (Table 2) and antiviral activity (Table 3).

**TABLE 3:** Antiviral activity of the tested nanomaterials/composites.

	Cell line L929 infected with the virus MHV-3	
**Samples**	**Reduction of viral infection in %**	**Effects**
AgNP@HST	99.99 ± 0.004	Virucide
GO	99.99 ± 0.004	Virucide
GO@AgNP@HST	99.99 ± 0.002	Virucide
CNF@GO@AgNP@HST	99.99 ± 0.005	Virucide

AgNP@HST, bio-based silver nanoparticles; GO, graphene oxide; GO@AgNP@HST, nanocomposites obtained using graphene oxide decorated with the AgNP@HST; CNF@GO@AgNP@HST, 3D nanocomposites obtained using CNF, GO, and AgNP@HST.

The samples were mixed with viruses and inoculated in the permissive cell line. Upon entering the cell successfully, the virus produces a cytopathic effect characterized by the production of syncytia and destruction of the cohesion of the cell monolayer. After 1 h of incubation, all tested materials provided a reduction of viral activity, in a standardized unit, of 99.99%, as shown in Table 3.

**FIGURE 2:**
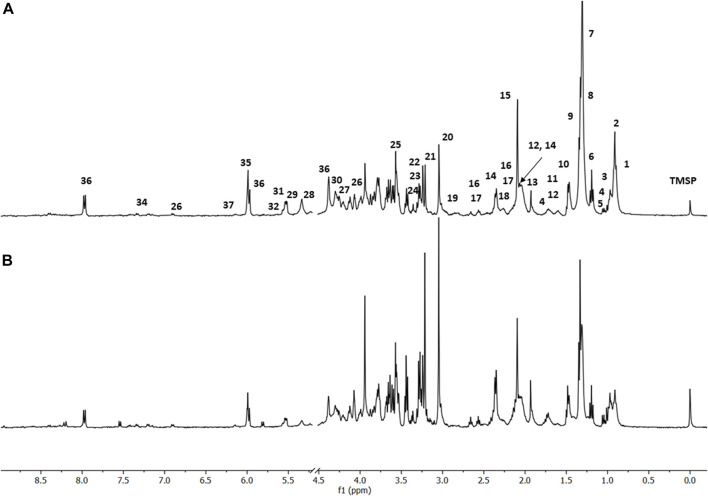
HR-MAS spectra showing: **(A)** ^1^H-NMR CPMG of fibroblast cells L929, with the exclusion of HDO, 4.80 ppm, **(B)** ^1^H-NMR CPMG of fibroblast cells L929 treated with the AgNP@HST. Spectra were acquired with Bruker AVANCE III 400 MHz at 25° C. For the metabolites’ assignments, see Supplementary Tables S1, S2.

**FIGURE 3:**
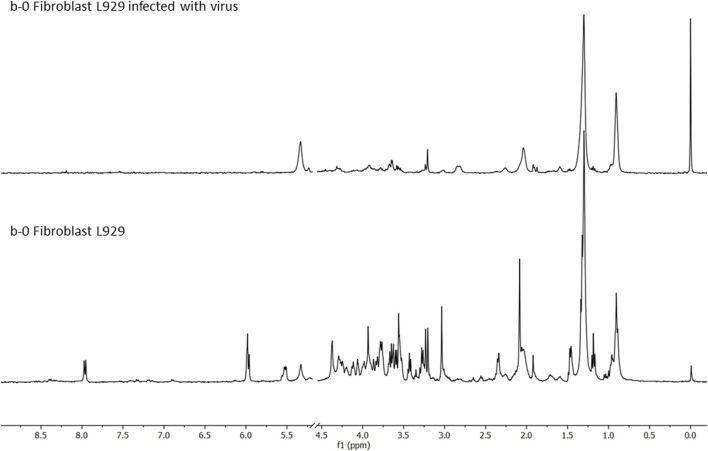
^1^H-NMR CMPG HR-MAS spectra of the fibroblast cells infected with the virus (MHV-3—upper panel, b-0 Fibroblast L929 infected with the virus), and the healthy fibroblast cells (b-0 Fibroblast L929), acquired using Bruker AVANCE III 400 MHz at 25° C, from 0.5 to 9.0 ppm (with the exclusion of HDO, 4.8 ppm).

**FIGURE 4:**
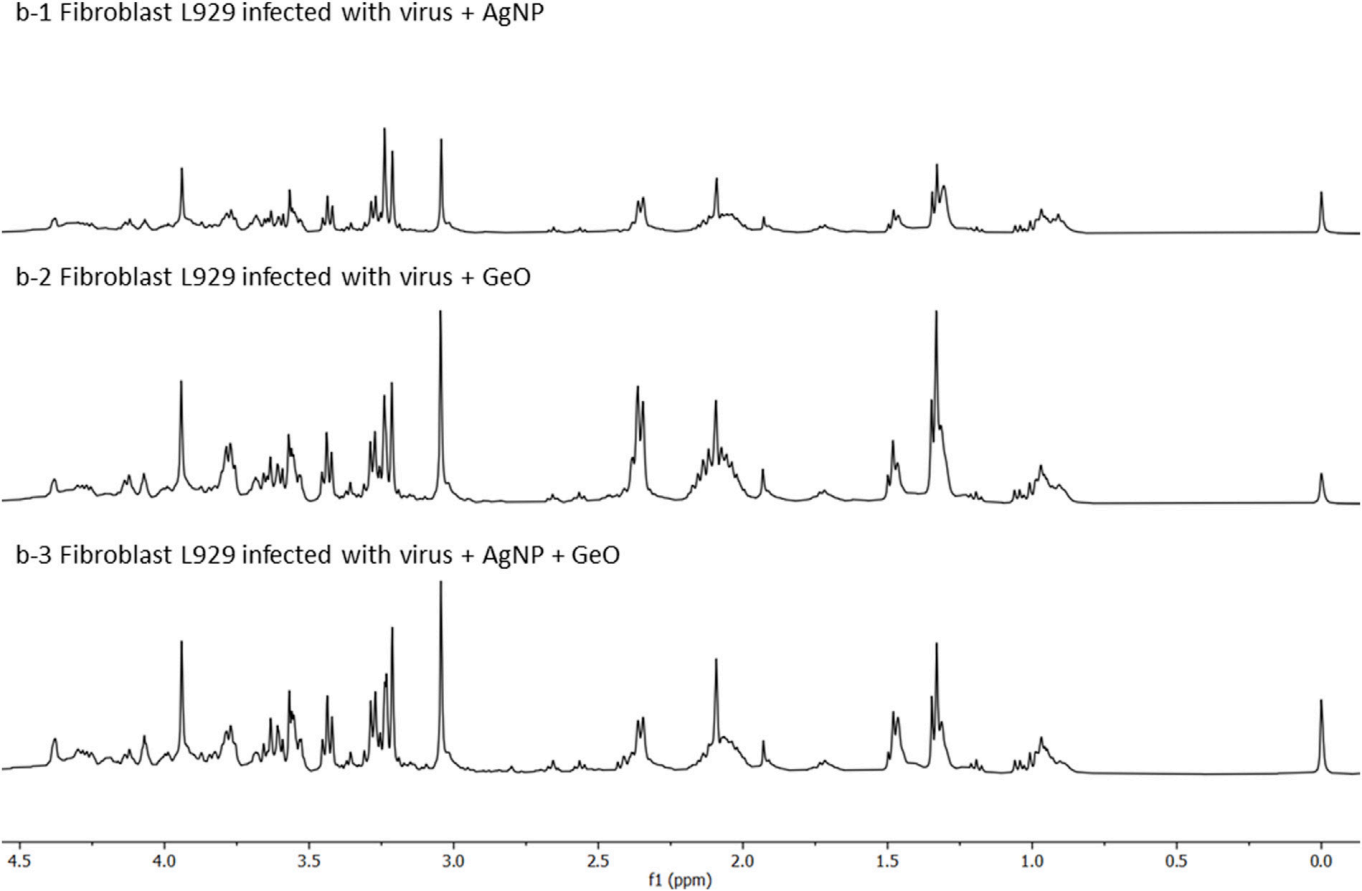
HR-MAS ^1^H-NMR spectra in 0.0–4.5 ppm region. Charts depict, from top to bottom: (b-1) infected fibroblast L929 cells with the virus MHV-3 in the presence of AgNP@HST (dilution 1:10, v v^−1^), (b-2) infected fibroblast L929 cells with the virus MHV-3 in the presence of GO (dilution 1:10, v v^−1^), and (b-3) infected fibroblast L929 cells with the virus MHV-3 in the presence of GO@AgNP@HST (dilution 1:10, v v^−1^).

The following conditions were investigated for the HR-MAS ^1^H-NMR experiments: 1) culture of L929 cells as a comparison standard (Figure 2); 2) culture of L929 cells infected with the virus MHV-3 (Figure 3); 3) culture of L929 cells with the MHV-3 virus and AgNP@HST (1:100, v v^−1^) and 4) culture of L929 cells with the MHV-3 virus and GO@AgNP@HST (1:10, v v^−1^). The HR-MAS ^1^H-NMR spectra are shown in Figures 2–4, while the spectra of the last two conditions are shown in Figure 4. The addition of nanomaterials was the same for healthy or infected cell sample treatments, using concentrations that inhibit the spread of the MHV-3 virus but do not disrupt the L929 cells as can be seen in Figure 2, where the identified metabolites in the healthy cells (1–36) were also present in the cells that were treated with AgNP@HST just to exemplify nanomaterial effects on cells.

The viral effects were devastating regarding cell metabolism, as shown in Figure 3 and Supplementary Table S2. There were observed many spectral differences in cells metabolites, mostly in the following spectral regions: (I) 0.8–2.4 ppm assigned to lipids, being more pronounced in the cells infected with the virus; (II) 3.0–4.5 ppm, pointing to glucose and amino acids decrease in the infected cells, and (III) 5.5–9.0 ppm presenting almost no picks for the infected cells. The lipid, fatty acids aliphatic chains (-CH_3_ and -CH_2_-), cholesterol (-CH_3_ group), cholesterol esters (-CH_3_ group), then, unsaturated fatty acids (-CH = CH-) loads showed increases in the MHV-3 infected cells, while all other metabolites showed decreases (Supplementary Table S2). The infected cells suffered changes in anabolic pathways that led to fatty acids, cholesterol, and lipids’ syntheses (phospholipids, PL), which were strongly activated, because of the viral replication and the need to construct (synthesize) and organize (build) new viral nanostructures, principally made from PL, RNA, and proteins. Intense nucleotide biosynthesis and alterations in the pentose phosphate pathway were also observed in the samples of MHV-3 infected cells with consequent RNA synthesis for the newly synthesized viral particles (MHV-3 genetic load). Almost all cell metabolites from the TCA cycle were depleted in the infected cells. Other metabolic pathways were activated in the infected cells, such as glutaminolysis, and glycolysis which depleted amino acids and glucose cells’ levels, which were explored for the viral proteins’ synthesis and energy requirements for intensive biosynthetic processes, respectively. These alterations in metabolism were expected, as infected cells use metabolic responses to survive, and adapt their metabolism by exploring alternative supplies for survival, such as for the TCA cycle, for example, by supplying glutamine to the TCA cycle. Also, a decrease in glutathione concentration was measured in the MHV-3 infected cells samples, which points to oxidative stress, probably caused by the intensive anabolic activity.

The observed harmful viral effects were reverted when cells were treated with the designed nanomaterials. Cell metabolomic profiles (Figure 4) showed that there are no evident differences between the cell metabolites, after infection when treated with AgNP@HST (Figure 4, b-1). The infected cells with the addition of other nanomaterials, namely GO (Figure 4, b-2), and GO@AgNP@HST (Figure 4, b-3), showed the same sets of cells metabolites in comparison to the cells grown in a healthy environment during the culture growth, indicative of the preserved metabolism despite the viral loads. It was seen that glucose, amino acids (alanine, glutamate, glutamine, leucine, valine, isoleucine, threonine, lysine, tyrosine, and phenylalanine, Supplementary Table S1), lipids, nucleotides, pentose phosphate pathway metabolites, then, choline, phospholipids, cholesterol, cholesterol esters, and lipids showed concentration levels equal or similar to ones measured for the healthy cells. Therefore, the cells re-established their metabolism when the infected cells were treated with the nanomaterials. These findings indicate that the designed nanomaterials had no toxic effects on the cells and that the viral MHV-3 infection effects were reverted, pointing to the nanomaterials’ strong antiviral activity.

## Discussion

This work aimed at the design and development of biocompatible virucidal materials, such as bio-based silver nanoparticles, and 3D flexible nanostructure composite materials, to be successfully used against coronaviruses. Silver nanoparticles have been prepared using hesperetin, a citrus fruit bioflavonoid. The design of hesperetin coatings on the silver(0) surface targeted the formation of highly biocompatible nanoparticles with enhanced virucidal effects. The one-step synthesis, completed in a few seconds, lead to stable colloids, with relatively uniform and highly dispersed AgNP@HST. It is important to highlight that it is the first time silver nanoparticles are obtained using hesperetin as the reducing and stabilizing agent. The AgNP@HST can be used as prepared for direct applications on surfaces and sprays for topical use (eyes, skin), nasal or mouth rinses, or as antiviral agents decorated onto 3D flexible nanostructured materials.

Cellulose nanofibers prepared from orange peels were used as hydrophilic and resistant matrices which, together with the graphene oxide, enabled to fabrication of flexible 3D nanostructures, further decorated with the AgNP@HST. This is also the first flexible nanomaterial made from orange peel cellulose nanofibers that are highly water-absorptive, up to six-fold superior when compared to cotton. As drops, droplets, and aerosol derived from the infected person’s saliva play a crucial role in the transmission of severe acute respiratory syndrome (SARS-CoV-2), the designed flexible 3D nanomaterials can provide an easy passage for purifying air, absorbing water, and neutralizing or binding to the viral particles (Supplementary Figue S6). It is important to mention that the viral particles are around 90–110 nm large and might interact with the AgNP@HST or GO or CNF. As the AgNP@HST particles are placed on the surface of the material and within its thin layer, they are the first to interact with the virus, followed by graphene oxide and CNF.

The MHV-3, prototype group II coronavirus, has been used as a model for the study of coronavirus replication and pathogenesis as exhibits great similarities with the SARS-CoV-2 virus. The viral infection changed fibroblast cells’ metabolites, principally in two spectral regions, lipids (more pronounced in the infected cells) and amino acids. Infected cells did not show 5.98 and 7.97 ppm peaks, which correspond to the nucleotides UDP and UTP. Also, peaks at 5.53 and 5.57 ppm that correspond to the multiples of *N*-acetylglucosamine and *N*-acetylgalactosamine were not found (Supplementary Tables S1, S2). These metabolites correspond to amino-, nucleotide sugar metabolism, and pyrimidine metabolism. O-*N*-Acetyl-D-glucosamine regulates cellular responses such as protective response to stress, modulates cell growth and division, and regulates gene transcription. Besides, O-*N*-acetyl-D-glucosamine-modified proteins are involved in sensing the nutrient status of the surrounding cellular environment and adjusting the activity of cellular proteins accordingly. A single *N*-acetylglucosamine moiety, linked to serine or threonine residues on nuclear and cytoplasmic proteins (-O-GlcNAc), is a ubiquitous post-translational protein modification. Nine sugar nucleotides can be classified depending on the type of nucleoside forming them: UDP-Plc, UDP-Gal, UDP-GlcNAc, UDP-GlcUA, UDP-Xyl, GDP-Man, GDP-Fuc, and CMP-NeuNAc. Observing the nucleotide sugar metabolism pathways, it should be noted that the nucleotide sugars play important roles as donors of important residues of sugar, vital to glycosylation, and polysaccharides’ synthesis. The ability of HR-MAS ^1^H-NMR to explore the metabolic status within healthy and virus-infected cells helped to understand the physiological and pathological transformations provoked by the virus (Supplementary Tables S2, S3). Consequently, detailed insights into the metabolic mechanisms reverted with the use of the designed nanomaterials, were provided. Furthermore, it was observed that none of the tested nanomaterials were toxic to cells, at least under the investigated conditions and quantities.

The mechanisms of action of AgNP@HSP can be linked to Ag (0) and Ag (I) joint actions, by probable AgNP@HST oxidation to Ag (I), and then by Ag (I) ions interactions with available biomolecules. As shown in Supplementary Figure S6, AgNP@HST surrounded SARS-CoV-2 viral particles (TEM images), and the viral proteins and PL are the first targets when considering the antiviral effect of AgNP@HST.

## Conclusion

The bio-based and green silver nanoparticles (AgNP@HST) were successfully synthesized using hesperetin as a reductive and stabilizing agent. The nanoparticles exhibited a mean diameter size of 22 nm, low zeta potential (−40 mV), and stability for at least 24 months. The nanoparticles showed spherical morphology, the predominance of Ag(0), and excellent virucidal effects against coronavirus. The AgNP@HST were used to decorate two 3D flexible and nanostructure materials graphene oxide, and composite obtained from cellulose nanofibers (CNF) and GO. Both nanomaterials showed high surface area, and good mechanical properties, while immobilization of AgNP@HST occurred on the surface of GO. The nanomaterials were cell-friendly, as cells maintained their morphology, growth rates, and intact metabolism according to the HR-MAS ^1^H-NMR analyses. Our results strongly suggest that all designed materials, especially AgNP@HST and CNF@GO@AgNP@HST, can stop the spread of the virus since, for all *in vivo* tests performed using virus-infected cells, these tailored materials blocked the virus and did not harm the cells. Finally, it is very important to state that none of the designed and fabricated nanomaterials were toxic to the fibroblast cells, thus being safe for use in medical applications. The presented results are important, as they can assist in the management of coronaviruses, which can provoke pandemics and millions of deaths in a short time. The use of the fabricated 3D materials is promising for air sterilization, principally in the medical environment, and sanitization purposes.

### Strengths and Limitations

There are some limitations of the current study such as still limited knowledge on a molecular basis regarding mechanisms involved in antiviral effects of the studied nanomaterials, although the cell infection with the virus was evaluated with success, and also materials’ effects on cells and cells infected with the virus were thoroughly investigated.

### Future Perspectives

Work is now in progress for the characterization of the CNF@GO@AgNP@HST composites regarding their porosity and permeability to better evaluate their filtering performance. Materials’ reuse, re-activation, recycling, and also, thermal, and structural properties of 3D materials are underway.

## Data Availability

The original contributions presented in the study are included in the article/Supplementary Material, further inquiries can be directed to the corresponding author.
