# Supplementary material for: High-Resolution Magic-Angle Spinning NMR Spectroscopy for Evaluation of Cell Shielding by Virucidal Composites Based on Biogenic Silver Nanoparticles, Flexible Cellulose Nanofibers and Graphene Oxide
Source: Front Bioeng Biotechnol. 2022 May 12;10:858156. doi: 10.3389/fbioe.2022.858156 (PMC9133937; doi:10.3389/fbioe.2022.858156)
Supplement: Supplementary file 1 [file DataSheet1.PDF]

Supplementary Material

High-Resolution Magic-Angle Spinning NMR Spectroscopy for evaluation of cell shielding by virucidal composites based on biogenic silver nanoparticles, flexible cellulose nanofibers and graphene oxide

Danijela Stanisic[1], Guilherme C. F. Cruz[1,2,3], Leonardo Abdala Elias[3,4], Junko Tsukamoto[5], Clarice W. Arns[5], Douglas Soares da Silva[6], Stanislav Mochkalev[2], Raluca Savu[2], Ljubica Tasic[1]***

[1]Chemical Biology Laboratory, Institute of Chemistry, Department of Organic Chemistry, University of Campinas (UNICAMP), Campinas, São Paulo, Brazil

[2]Centre for Semiconductor Components and Nanotechnology (CCSNano), University of Campinas (UNICAMP), Campinas, São Paulo, Brazil

[3]Center for Biomedical Engineering (CEB), University of Campinas (UNICAMP), Campinas, São Paulo, Brazil

[4]Department of Electronics and Biomedical Engineering, School of Electrical and Computer Engineering, University of Campinas (UNICAMP), Campinas, São Paulo, Brazil

[5]Laboratory of Animal Virology, Institute of Biology, Department of Genetics, Evolution, Microbiology, and Immunology, University of Campinas (UNICAMP), São Paulo, Brazil

[6]Institute of Chemistry, University of Campinas (UNICAMP), Campinas, São Paulo, Brazil

*** Correspondence:**
Ljubica Tasic
ljubica@unicamp.br

Keywords: Silver nanoparticles, hesperetin, graphene oxide, cellulose nanofibers (CNF), composites, virus

*****In memoriam:** We dedicate this work to our dear colleague, Douglas Silva, who passed away at the end of 2021, at the young age of 38. We will miss you!*

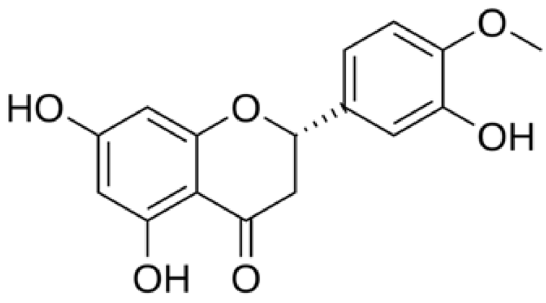

(a)

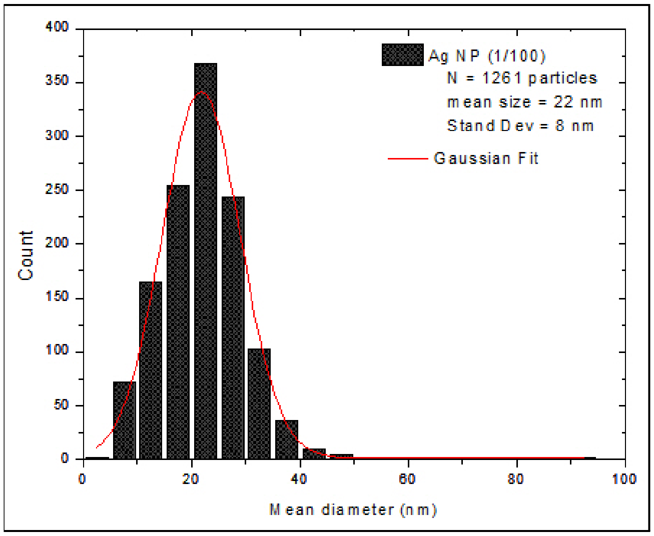

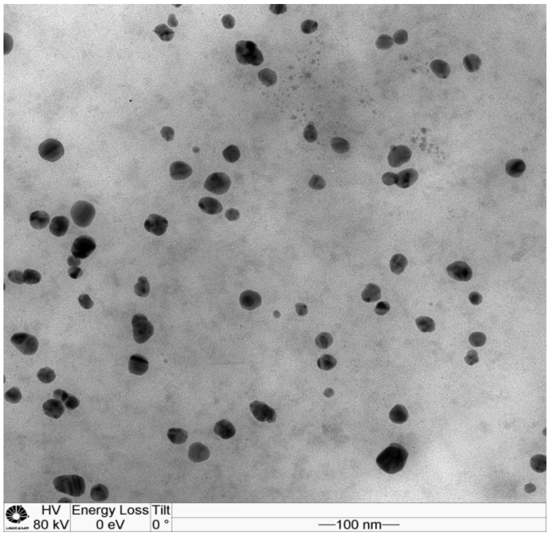

(b) **(c)**

Supplementary Figure SF1. (a) Structure of hesperetin. (b) AgNP@HST showed mean diameters of 22 ± 8 nm, (c) spherical forms (scale bar = 100 nm), PDI lower than 0.24, and zeta potential of -40 ± 1 eV.

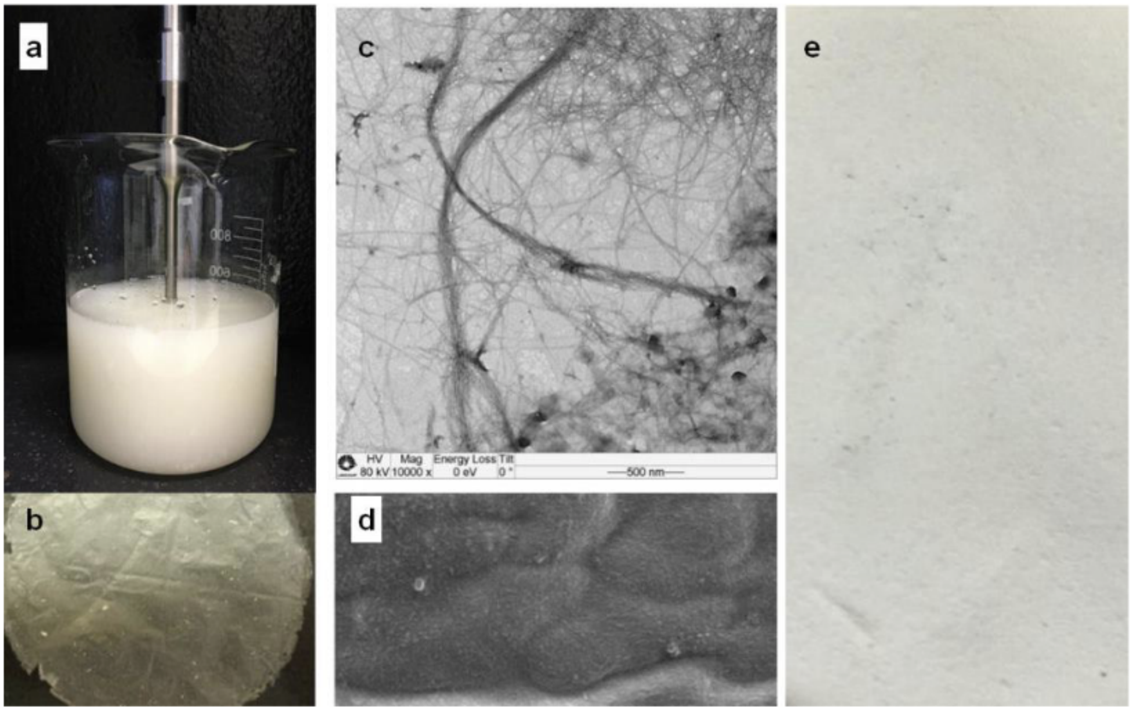

Supplementary Figure SF2. Cellulose nanofibers (CNF) produced from orange bagasse were used for the fabrication of flexible 3D composite nanomaterials because of their excellent mechanical properties such as strength. (a) Suspension of CNF in NaOH (7%), (b) thin paper produced from CNF; (c) TEM image of CNF (scale bar = 500 nm); (d) SEM image of the paper shown in b (scale bar = 1000 nm); and (e) CNF paper under microscope lenses.

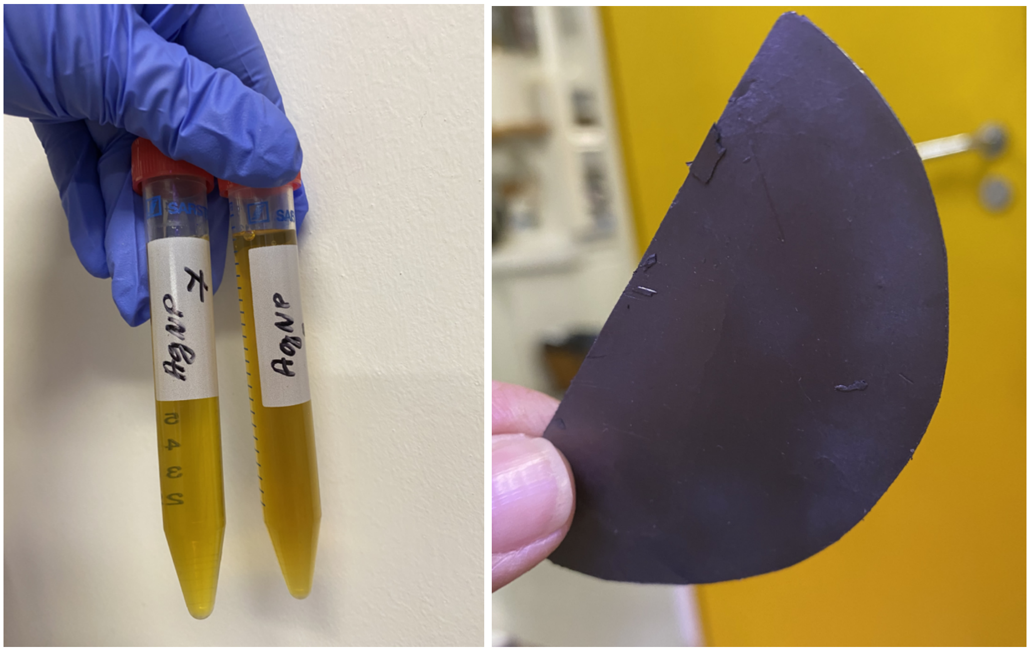

Supplementary Figure SF3. Photographs of the synthesized silver nanoparticles AgNP@HST colloid (left) and flexible 3D composite nanomaterial fabricated from CNF, graphene oxide, and decorated with the synthesized, namely CNF@GO@AgNP@HST (right).

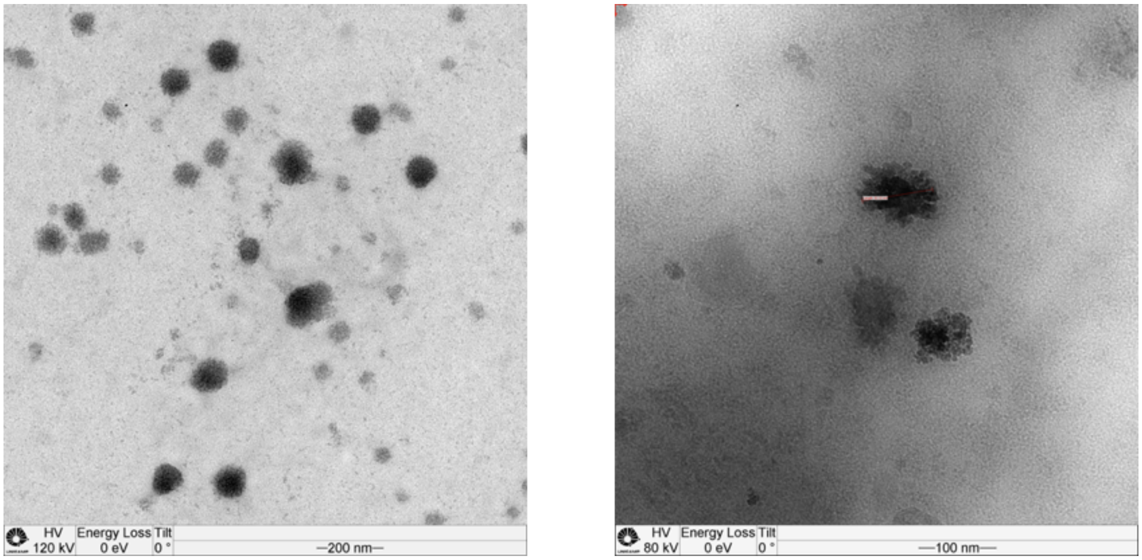

Supplementary Figure SF4. Transmission electron micrographs of coronaviruses - MHV-3 (left - scale bar = 200 nm) and SARS-CoV-2 (right - scale bar = 100 nm). The studied viruses are very similar in size and form.

a

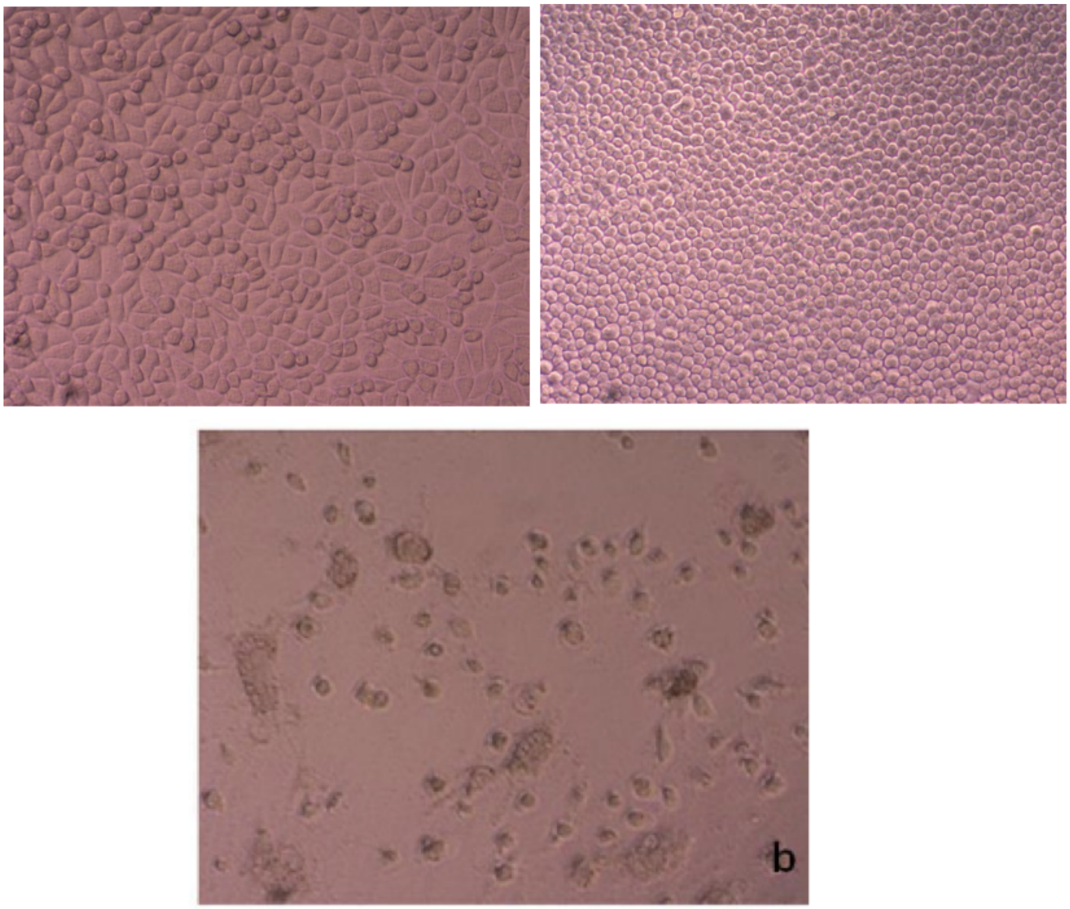

Supplementary Figure SF5. Photographs showing microscopic views onto fibroblast cells: (**a**) cultured in rich medium (upper panel left), and treated with the synthesized AgNP@HST (upper panel right), and (**b**) infected with MHV-3,

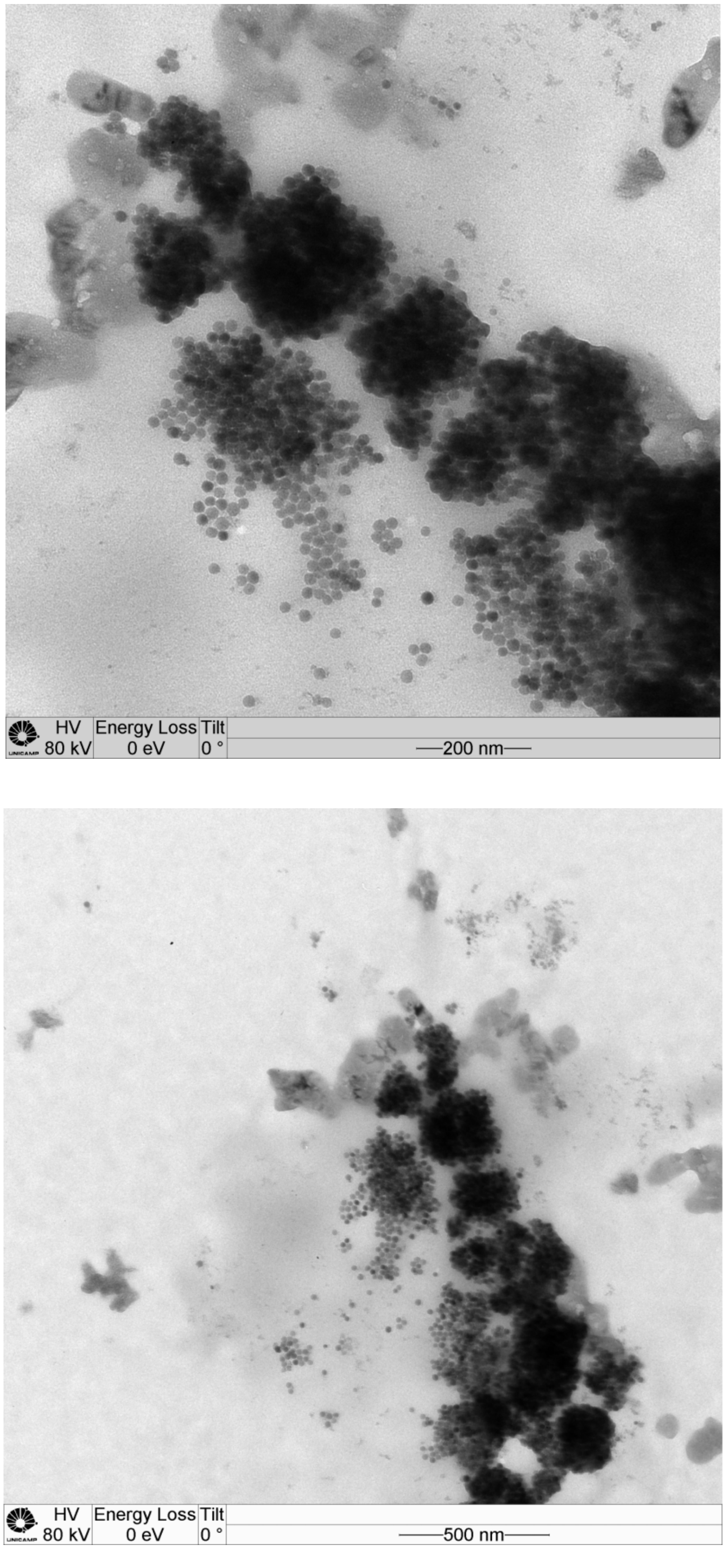

Supplementary Figure SF6. Transmission electron micrographs showing interaction between SARS-CoV-2 virus and AgNP@HST (upper panel - scale bar = 200 nm and lower panel - scale bar = 500 nm). The viruses (around 100 nm) were totally covered with AgNP@HST.

Supplementary Table ST1. Main metabolites identified by HR-MAS ^{1}H-NMR in Fibroblast cells line L929 (see Figure 2)

Peak	Chemical shift [ppm]	Compound
1	0.723	Cholesterol & cholesterol esters (-CH_3)
2	0.893; 0.904	Lipids (-CH_3)
3	0.96; 0.97; 1.02	Isoleucine
4	0.97; 1.73	Leucine
5	1.03	Valine
6	1.19	Ethanol (residual)
7	1.29; 3.065	Threonine
8	1.30	Lipids
9	1.33; 4.12	Lactate
10	1.47; 1.72; 3.02	Lysine
11	1.48; 3.78	Alanine
12	1.60; 2.04	Lipids
13	1.98	Acetate
14	2.06; 2.36; 3.784	Glutamine
15	2.09	UDP-*N*-Acetyl-glucosamine /galactosamine
16	2.18; 2.57; 2.96; 3.928	Glutathione
17	2.18; 2.57; 3.8	Glutamate
18	2.25	Lipids
19	2.815	Lipids
20	3.05; 3.94; 4.20	Creatine
21	3.21; 3.53; 4.07	Choline
22	3.23; 3.6	Phosphocholine - Phospholipids (PL)
23	3.24; 4.00	Phosphoethanolamine (PL)
24	3.27; 3.68	Glycerophosphocholine
25	3.28; 3.55	Myo-inositol
26	3.96; 6.92; 7.21	Tyrosine
27	4.28	Ribose-5-phosphate
28	5.33	Lipids (- CH=CH-)
29	5.43	Glucosamine
30	5.46	Glucose-phosphate
31	5.55	UDP-*N*-acetyl-glucosamine
32	5.59	UDP-*N*-acetyl-galactosamine
33	5.67	UDP-Glucuronic acid
34	5.82; 7.55	Uracil
35	5.92; 7.86	Uridine
36	4.38; 5.98; 7.95	UDP/UTP
37	6.07; 7.84	Cytidine
38	7.35; 7.44	Phenylalanine
39	8.23; 8.58	AMP

Supplementary Table ST2. ^{1}H-NMR peaks' assignments in infected fibroblast cells,lipids were increased in HR-MAS NMR spectra of the MHV-3 infected cells

**NMR shifts (ppm)**	**Peak Intensities level change**	**Compound**
0.97; 1.73	↓ decrease	Leucine
1.47; 1.72; 3.02	↓ decrease	Lysine
1.48; 3.78	↓ decrease	Alanine
1.98	↓ decrease	Acetate
2.04; 5.30	↑ increase	Lipids
2.06; 2.36; 3.784	↓ decrease	Glutamine
2.09; 5.52	↓ decrease	UDP-*N*-Acetyl-glucosamine /galactosamine
2.18; 2.57; 2.96; 3.928	↓ decrease	Glutathione
2.18; 2.57; 3.8	↓ decrease	Glutamate
3.05; 3.94; 4.20	↓ decrease	Creatine
3.21; 3.53; 4.07	↓ decrease	Choline
3.23; 3.6	↓ decrease	Phosphocholine
3.27; 3.68	↓ decrease	Glycerophosphocholine
3.28; 3.55	↓ decrease	Myo-inositol
5.43	↓ decrease	Glucosamine
5.46	↓ decrease	Glucose-phosphate
5.55	↓ decrease	UDP-*N*-Acetyl-glucosamine
5.59	↓ decrease	UDP-*N*-Acetyl-galactosamine
5.98; 7.95	↓ decrease	UDP/UTP
7.35; 7.44	↓ decrease	Phenylalanine
7.86	↓ decrease	Uridine

Supplementary Table ST3. Results from Pathway Analysis alteration upon viral infection

Pathway Name	Match Status	p	-log(p)	Holm p	FDR	Impact
Aminoacyl-tRNA biosynthesis	6/48	4.069E-6	12.412	3.4179E-4	3.4179E-4	0.0
Nitrogen metabolism	2/6	0.0014636	6.5269	0.12148	0.04098	0.0
D-Glutamine and D-glutamate metabolism	2/6	0.0014636	6.5269	0.12148	0.04098	0.5
Alanine, aspartate and glutamate metabolism	3/28	0.0025282	5.9802	0.20479	0.053092	0.3109
Glyoxylate and dicarboxylate metabolism	3/32	0.003732	5.5908	0.29856	0.062698	0.0
Glycerophospholipid metabolism	3/36	0.0052379	5.2518	0.41379	0.07333	0.08333
Pyrimidine metabolism	3/39	0.006578	5.024	0.51309	0.078936	0.05274
Arginine biosynthesis	2/14	0.0084606	4.7723	0.65147	0.088836	0.11675
Glutathione metabolism	2/28	0.032306	3.4325	1.0	0.30152	0.27562
Phenylalanine, tyrosine, and tryptophan biosynthesis	1/4	0.040694	3.2017	1.0	0.33442	0.5
Glycine, serine, and threonine metabolism	2/33	0.043793	3.1283	1.0	0.33442	0.0
Arginine and proline metabolism	2/38	0.056589	2.8719	1.0	0.39612	0.09812
Ascorbate and aldarate metabolism	1/8	0.079832	2.5278	1.0	0.47899	0.0
Valine, leucine, and isoleucine biosynthesis	1/8	0.079832	2.5278	1.0	0.47899	0.0
Biotin metabolism	1/10	0.098834	2.3143	1.0	0.51888	0.0
Phenylalanine metabolism	1/10	0.098834	2.3143	1.0	0.51888	0.35714
Butanoate metabolism	1/15	0.14474	1.9328	1.0	0.71521	0.0
Histidine metabolism	1/16	0.15366	1.873	1.0	0.71707	0.0
Selenocompound metabolism	1/20	0.18845	1.6689	1.0	0.79151	0.0
Ether lipid metabolism	1/20	0.18845	1.6689	1.0	0.79151	0.0

Since we were testing many pathways at the same time, the statistical p-values from enrichment analysis are further adjusted for multiple testing. Thus, **Total** is the total number of compounds in the pathway; the **Hits** is the matched number from the user uploaded data; the **Raw p** is the original

p-value calculated from the enrichment analysis; the **Holm p** is the p-value adjusted by the Holm-Bonferroni method; the **FDR p** is the p-value adjusted using False Discovery Rate; the **Impact** is the pathway impact value calculated from pathway topology analysis.
